# Oxygenation strategies after extubation of critically ill and postoperative patients

**DOI:** 10.1016/j.jointm.2021.05.003

**Published:** 2021-06-29

**Authors:** Arnaud W. Thille, Mathilde Wairy, Sylvain Le Pape, Jean-Pierre Frat

**Affiliations:** 1 Médecine Intensive Réanimation, Centre Hospitalier Universitaire de Poitiers, Poitiers 86021, France; 2 ALIVE Research group, INSERM CIC 1402, University of Poitiers, Poitiers 86021, France

**Keywords:** Noninvasive ventilation, High-flow nasal oxygen therapy, Ventilator weaning, Airway extubation, Mechanical ventilation, Intensive care unit

## Abstract

In intensive care units (ICUs), the decision to extubate is a critical one because mortality is particularly high in case of reintubation. Around 15% of patients ready to be weaned off a ventilator experience extubation failure leading to reintubation. The use of high-flow nasal oxygen and non-invasive ventilation are two alternatives of standard oxygen supplementation that may help to prevent reintubation. High-flow nasal oxygen and non-invasive ventilation, may be used to prevent reintubation in patients with low (e.g., patients without comorbidities and with short durations of mechanical ventilation) and high risk (e.g., patients >65 years and those with underlying cardiac disease, chronic respiratory disorders, and/or hypercapnia at the time of extubation) of reintubation, respectively. However, non-invasive ventilation used as a rescue therapy to treat established post-extubation respiratory failure could increase mortality by delaying reintubation, and should therefore be used very carefully in this setting. The oxygenation strategy to be applied in postoperative patients is different from the patients who are extubated in the ICUs. Standard oxygen after a surgical procedure is adequate, even following major abdominal or cardiothoracic surgery, but should probably be switched to high-flow nasal oxygen in patients with hypoxemic. Unlike in patients experiencing post-extubation respiratory failure in ICUs wherein non-invasive ventilation may have deleterious effects, it may actually improve the outcomes in postoperative patients with respiratory failure. This review discusses the different clinical situations with the aim of choosing the most effective oxygenation strategy to prevent post-extubation respiratory failure and to avoid reintubation.

## Introduction

In intensive care units (ICUs), the decision to extubate is a critical one, because mortality is particularly high in patients who require reintubation [1], [2], [3]. Despite compliance with all intubation weaning criteria and successful trial of spontaneous breathing, the overall rate of reintubation after a planned extubation is still around 15%, which may exceed 20% in high-risk patients [4,5]. In clinical practice, the majority of patients extubated in the ICUs are treated with standard oxygen, and 10–20% of patients receive either non-invasive ventilation or high-flow nasal oxygen after extubation [6], [7], [8]. In patients at low risk of extubation failure, high-flow nasal oxygen is an alternative strategy that may reduce the risk of reintubation as compared with standard oxygen therapy [9,10]. In patients at high risk of extubation failure, several clinical trials have shown that compared with standard oxygen, non-invasive ventilation may decrease post-extubation respiratory failure and the need for reintubation [11], [12], [13], [14]. The most recent international clinical practice guidelines recommend the use of non-invasive ventilation in this population [15]. Recently, a large clinical trial showed that compared with high-flow nasal oxygen, non-invasive ventilation alternating with high-flow nasal oxygen significantly reduced the risk of reintubation in patients with high-risk of extubation failure, further reinforcing the beneficial effects of non-invasive ventilation [16]. Although the criteria to identify the patients at high risk of extubation failure differ across the studies, they were easily identified in the latter trial as being patients > 65 years or those with underlying chronic cardiac or lung disease [16,17]. Thereby, high-flow nasal oxygen alone might be the most effective oxygenation strategy for patients at low risk of extubation failure, while non-invasive ventilation alternating with high-flow nasal oxygen could be the most effective strategy for patients at high risk.

**Fig. 1:**
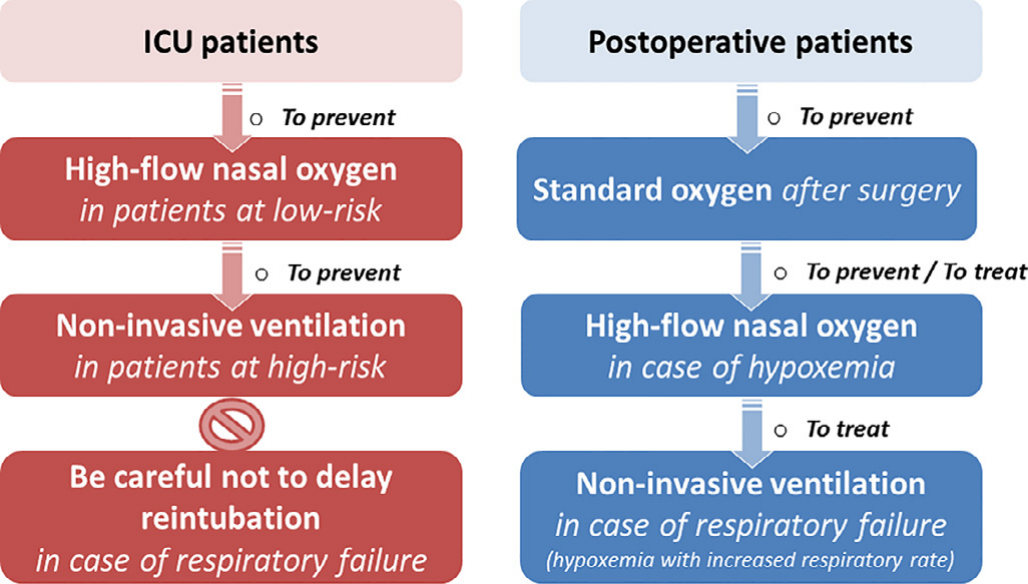
Proposal of management of oxygenation strategies to prevent or treat respiratory failure in patients extubated in ICUs and in postoperative patients.ICUs: Intensive care units.

The optimum oxygenation strategy for postoperative patients is different from that of extubated patients in the ICU [Fig. 1]. While several studies have compared high-flow nasal *vs.* standard oxygen in postoperative patients, the beneficial effects of high-flow nasal oxygen have not been clearly demonstrated and standard oxygen remains the reference treatment [18], [19], [20]. Management of postoperative patients extubated after a few hours of mechanical ventilation following a surgical procedure should be distinguished from that of patients mechanically ventilated for several days in the ICU. However, a number of postoperative patients need prolonged duration of mechanical ventilation due to complications, especially after an urgent surgery, and around 20% of patients extubated in ICUs are originally intubated for surgical procedures [5,6]. These patients should be considered as patients at high risk of extubation in ICUs and not as postoperative cases [11,^16^,^21^,22]. Consequently, they should preferentially receive an oxygenation therapy suited for patients extubated in the ICU and not that suited for postoperative patients.

## Oxygenation Strategies in Patients Extubated in the ICUs

### **Prevention of reintubation in patients at high risk of extubation failure: Non-invasive ventilation, high-flow nasal oxygen, or both?**

**Fig. 2:**
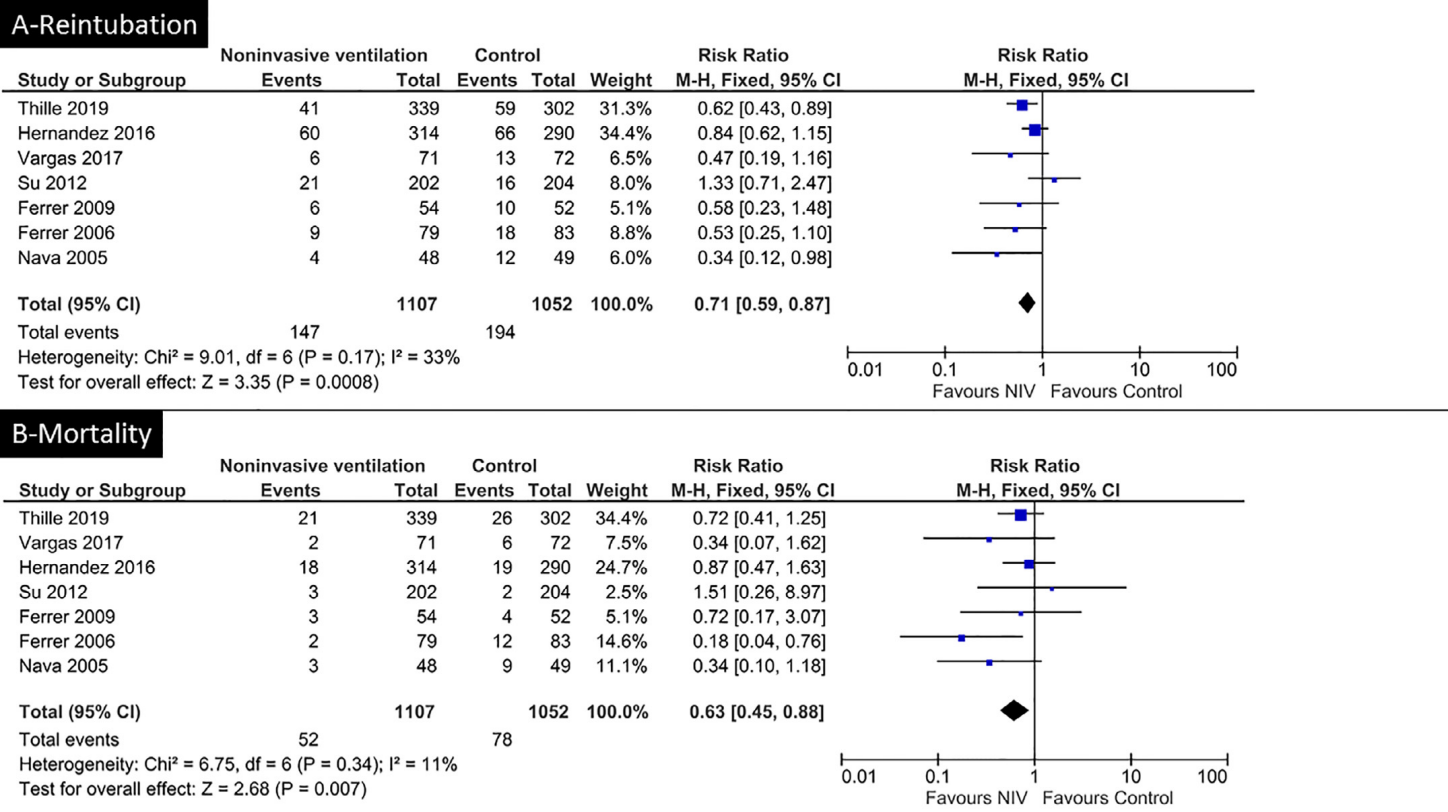
Effect of non-invasive ventilation on reintubation rate (A) and on mortality rate (B) when compared with standard oxygen or high-flow nasal oxygen. By pooling the main multicenter, randomized controlled trials, the prophylactic use of non-invasive ventilation immediately after extubation is associated with reduced risk of reintubation (A) and decreased risk of death (B) in ICU. ICU: Intensive care unit.

The most recent international clinical practice guidelines recommend the use of non-invasive ventilation after extubation in patients at high risk of reintubation [15]. Indeed, several randomized controlled trials have shown that compared with standard oxygen, non-invasive ventilation may decrease post-extubation respiratory failure, reintubation, and even risk of death [11], [12], [13], [14]. Pooling all multicenter randomized controlled trials that compared non-invasive ventilation *vs*. standard oxygen or high-flow nasal oxygen after extubation in ICU showed that non-invasive ventilation likely decreases the risk of reintubation and the risk of death, when compared with other oxygenation strategies [11], [12], [13], [14],^16^,^21^,22] [Fig. 2]. In some studies, non-invasive ventilation has been applied continuously [12,^14^,23] or intermittently [11,13] within the first 24 h or the first 48 h after extubation, up to 8–18 h per day. Although the criteria for high risk of extubation failure have differed among trials, most of these studies included patients > 65 years and those with chronic respiratory disorders, underlying cardiac disease, several comorbidities, prolonged duration of mechanical ventilation with difficult weaning, or hypercapnia at the time of extubation [11], [12], [13], [14],^16^,21]. Non-invasive ventilation could be particularly effective in preventing reintubation among patients with weak cough [24]; furthermore, patients with the inability to clear secretions have also been considered as high risk for extubation failure in several clinical trials [11,21]. While the majority of patients were initially intubated for acute respiratory failure, a number of them had been intubated for surgery or trauma management.

Other trials have compared the prophylactic use of non-invasive ventilation *vs.* standard oxygen in patients extubated after at least 48 h or 72 h of mechanical ventilation and those who were not necessarily considered at high risk of extubation failure [22,^23^,25]. Two of these studies also showed a reduced risk of extubation failure knowing that >30% of patients had underlying cardiac or respiratory disease [23,25]. In all the above-mentioned studies, reintubation rates in the control group treated with standard oxygen exceeded the usual 15% rate, suggesting that these patients were actually at high risk of extubation failure. Finally, a large-scale randomized controlled trial comparing non-invasive ventilation *vs.* standard oxygen in patients intubated for at least 48 h reported reintubation rates that were strictly similar (about 10%) in both groups [22], suggesting that non-invasive ventilation may be not effective in patients with low reintubation rates, e.g.*,* lower than the usual threshold of 15% in the control group.

In a large-scale randomized clinical trial including 604 patients with high risk of extubation failure, Hernandez et al. [21] reported that high-flow nasal oxygen was non-inferior to non-invasive ventilation in preventing reintubation. Since non-invasive ventilation could be particularly effective in patients with hypercapnic (PaCO_2_ >45 mmHg) [12,26], they were excluded at the time of extubation. The rate of reintubation 72 h after extubation was 19% with non-invasive ventilation and 23% with high-flow nasal oxygen alone, and the authors concluded that high-flow nasal oxygen alone could be enough to manage patients  without hypercapnia at high risk of extubation failure in the ICU [21]. Although high-flow nasal oxygen could be considered as a reference treatment after extubation, its association with non-invasive ventilation may further improve gas exchange and the work of breathing [27]. In another multicenter, randomized clinical trial involving 641 patients at high risk of extubation failure in the ICU, e.g.*,* intubated for at least 24 h and > 65 years or with underlying chronic cardiac or lung disease, Thille et al. [16] compared high-flow nasal oxygen alternating with non-invasive ventilation *vs.* high-flow nasal oxygen alone. The proportion of patients who developed post-extubation respiratory failure or required reintubation was significantly lower with non-invasive ventilation alternating with high-flow nasal oxygen than with high-flow nasal oxygen alone. Although the reduction in the absolute risk of reintubation was greater in patients with hypercapnia than in patients without hypercapnia, the rate of reintubation in the ICU was significantly lower with non-invasive ventilation than with high-flow nasal oxygen alone, even in patients without hypercapnia (13% *vs.* 19%; difference, −6.6% [95% CI: −12.9% to −0.4%]; *P* *=* 0.037) [16]. Although the most recent international clinical practice guidelines recommend non-invasive ventilation after extubation in patients at high risk of extubation failure in ICUs, the strength of this recommendation was only conditional given the moderate certainty of evidence [15]. The results of this clinical trial further reinforce certainty and likely contribute to establishing strong recommendations on the use of preventive non-invasive ventilation after extubation in patients at high risk of reintubation in ICUs [Fig. 1]. Although high-flow nasal oxygen between sessions of non-invasive ventilation may be a particularly effective oxygenation strategy in patients at high risk of extubation failure, the use of high-flow nasal oxygen in this setting has not yet been demonstrated.

### **Prevention of reintubation in patients at low risk of extubation failure: High-flow nasal oxygen or standard oxygen?**

Several clinical trials have shown that compared with standard oxygen, high-flow nasal oxygen may reduce the risk of reintubation in the ICU [9,10]. Maggiore et al. [9] randomly assigned 105 patients with hypoxemia at the time of extubation (PaO_2_/FiO_2_ ratio, ≤300 mm Hg) to receive high-flow nasal oxygen instead of standard oxygen with a Venturi mask during the first 24 h after extubation, and showed that reintubation rates were significantly lower with high-flow nasal oxygen than with standard oxygen (4% *vs.* 21%, *P* = 0.01). Later, a large-scale clinical trial comparing high-flow nasal oxygen *vs.* standard oxygen in 527 patients in the ICU having low risk of extubation failure also showed lower reintubation rates with high-flow nasal oxygen than with standard oxygen (5% *vs.* 12%, *P* = 0.004) [10]. Patients were considered to have low risk of extubation failure if they were < 65 years; without underlying conditions such as heart failure, chronic obstructive pulmonary disease, obesity, or other comorbidities; intubated for <7 days; extubated after the first spontaneous breathing trial, without hypercapnia at the time of extubation; without the risk of developing laryngeal edema, and were able to clear secretions. Patients eventually included in this trial were mainly those admitted to the ICU after surgery or trauma and those with a short duration of mechanical ventilation (median: 1–2 days); <3% of patients met the criteria for acute respiratory distress syndrome [10]. The major limitation of this trial was that the rate of reintubation was particularly high in the control group (12%), given that this population had a very low risk of extubation failure. Although an additional clinical trial may reinforce the level of certainty, high-flow nasal oxygen immediately after extubation in patients at low risk of extubation failure is a promising option to prevent reintubation.

To sum up the possible guidelines for management of patients extubated in an ICU, the literature suggests the use of high-flow nasal oxygen immediately after extubation in patients with low risk of extubation failure with moderate evidence-based certainty; whereas, the use of non-invasive ventilation for at least 24 h would be strongly recommended in patients with high risk of extubation failure [Fig. 1]. The use of high-flow nasal oxygen between non-invasive ventilation sessions might be associated to further prevent reintubation, without there being any certainty.

### **Treatment of post-extubation respiratory failure: What are the risks of using non-invasive ventilation?**

Non-invasive ventilation applied immediately after extubation may prevent post-extubation respiratory failure [11], [12], [13], [14],16]; however, non-invasive ventilation used as a rescue therapy to treat established post-extubation respiratory failure could increase mortality by delaying reintubation [28]. The largest clinical trial conducted to date including 221 patients with post-extubation respiratory failure showed greater mortality with non-invasive ventilation than with standard oxygen [28]. Although reintubation rates were strictly similar between the two groups (48%), the ICU mortality rate was higher in patients treated with non-invasive ventilation than in those treated with standard oxygen (25% *vs.* 14%, *P* = 0.048). The only difference was that the reintubation delay was markedly longer with non-invasive ventilation than with standard oxygen (median of 12 h *vs*. 2 h after the onset of respiratory failure), suggesting that non-invasive ventilation may worsen outcomes by masking respiratory severity and delaying reintubation. In another clinical trial including 81 patients with post-extubation respiratory failure, reintubation and mortality rates did not significantly differ between patients treated with non-invasive ventilation and those treated with standard oxygen [29]. A meta-analysis of these two studies showed no benefit of non-invasive ventilation compared with standard oxygen [30]. No further clinical trials have been performed; hence, the international clinical practice guidelines suggest that non-invasive ventilation should not be used in the treatment of patients with established post-extubation respiratory failure [15]. Although the majority of patients included in both clinical trials had hypercapnia at the onset of respiratory failure (mean PaCO2: >45 mmHg), few patients had underlying chronic lung disease (∼10%) [28,29], and consequently this recommendation may not apply to patients with chronic respiratory disorders. Indeed, non-invasive ventilation may avoid reintubation in several hypercapnic patients with underlying chronic lung disease [12,^14^,^31^,32]. However, nearly half of all patients who experience post-extubation respiratory failure in ICUs eventually require reintubation, with particularly high mortality (>30%) patients who require reintubation [4,^5^,^16^,21]. Consequently, non-invasive ventilation to treat post-extubation respiratory failure in patients in the ICU should be used very carefully and only in patients with underlying chronic lung disease, paying attention to not delay reintubation when the criteria for reintubation are met [Fig. 1].

Although the beneficial effects of high-flow nasal oxygen have been reported in the treatment of *de novo* acute respiratory failure [33], it has never been specifically assessed for the management of post-extubation respiratory failure. Several physiological studies have shown that high-flow nasal oxygen may help to reduce work of breathing and PaCO_2_ almost as effectively as non-invasive ventilation [27,^34^,35]. However, whether high-flow nasal oxygen is actually effective for the management of post-extubation respiratory failure has not yet been elucidated.

## Postoperative Patients

### **High-flow nasal oxygen or standard oxygen after a major surgery?**

This question is of major importance given the large number of patients worldwide undergoing surgery (especially major abdominal, cardiac and thoracic surgeries) every day. A recent systematic review and meta-analysis were conducted to assess whether routine use of high-flow nasal oxygen was superior to standard oxygen in preventing intubation in postoperative patients [36]. This meta-analysis reported an overall intubation rate of 4% with standard oxygen *vs.* 1% with high-flow nasal oxygen (*P* = 0.030). Although none of the 11 randomized controlled trials included in the systematic review reported a significant difference in intubation, the pooled results from the meta-analysis of these studies showed that compared with standard oxygen, high-flow nasal oxygen applied immediately after surgery significantly decreased the need for intubation [36]. Post-hoc subgroup analysis suggested that this effect was driven by patients who were obese or at high risk, thereby supporting the routine use of high-flow nasal oxygen in these patients. However, the intubation risk analysis was evaluated based on only 6 of of the 11 trials included in the systematic review. Several trials were absent from the meta-analysis, especially the large-scale study of Futier et al. [18] including 220 patients after major abdominal surgery. In their trial, the proportion of patients who required intubation was 6.5% (7/108 patients) with high-flow nasal oxygen and 3.6% (4/112 patients) with standard oxygen [18]. By adding this trial to the meta-analysis, high-flow nasal oxygen was no longer significantly associated with a decreased risk of reintubation when compared with standard oxygen, either in the overall population or in the subgroup of patients at high-risk [37]. In conclusion, current literature does not support the routine use of high-flow nasal oxygen in postoperative patients with expected intubation rates not exceeding around 5%; thus, standard oxygen remains the reference treatment to be started immediately after major surgery.

### **Non-invasive ventilation or high-flow nasal oxygen to treat respiratory failure in postoperative patients?**

Unlike patients who experience post-extubation respiratory failure in the ICU in whom non-invasive ventilation may have deleterious effects, non-invasive ventilation may improve outcomes in postoperative patients with respiratory failure [38,39]. This difference might be because of the cause of respiratory failure, which is most often atelectasis in postoperative patients [39] and most often because of the inability to clear secretions or respiratory acidosis in patients in ICU who frequently have major muscle weakness [21,40]. In the majority of cases, postoperative patients are extubated immediately after surgical procedure, e.g.*,* after only a few hours of mechanical ventilation; whereas patients in ICU are extubated after several days of mechanical ventilation favoring the occurrence of respiratory muscle weakness [40], [41], [42].

Postoperative patients who experience respiratory failure (e.g.*,* hypoxemia with increased respiratory rate and/or signs of respiratory distress) within hours or days following surgery have expected intubation rates of ∼50% with standard oxygen [38,39]. A large-scale, randomized controlled trial including 293 patients with acute respiratory failure within the 7 days following abdominal surgery showed that compared with standard oxygen, non-invasive ventilation significantly decreased the risk of intubation (46% *vs.* 33% at day 7, *P* = 0.030) [39]. A previous trial including 48 patients with respiratory failure following thoracic surgery showed that compared with standard oxygen, non-invasive ventilation was associated with decreased intubation rate (50% *vs.* 21%, *P* = 0.035) and even decreased risk of death [38]. Consequently, the international clinical practice guidelines suggest non-invasive ventilation to treat patients who experience respiratory failure after major surgery (conditional recommendation, moderate certainty of evidence) [15]. It is still unclear whether high-flow nasal oxygen in place of standard oxygen between non-invasive ventilation sessions is effective as it has never been investigated so far.

Another large-scale clinical trial including 209 patients with hypoxemia after abdominal surgery showed that compared with standard oxygen, non-invasive ventilation using continuous positive airway pressure through a helmet significantly reduced the risk of intubation [43]. It must be underlined that these patients did not actually have respiratory failure, and non-invasive ventilation was applied more as a preventive than a curative measure. Indeed, patients included in this trial had hypoxemia (PaO_2_/FiO_2_<300 mm Hg immediately after surgery), but without increased respiratory rate and any clinical signs of respiratory distress. Consequently, the rate of intubation in the control group was only 10%, *e*.g., markedly <50% intubation rate observed in postoperative patients with respiratory failure [38,39]. By contrast, the overall expected intubation rates did not exceed 5% in postoperative patients, even in patients at high risk [36]. To treat hypoxemia without actual respiratory failure, *e*.g., with expected intubation rates around 10–15%, high-flow nasal oxygen could be as effective as non-invasive ventilation [44]. Indeed, in a large-scale clinical trial including 830 patients with hypoxemia after cardiothoracic surgery, high-flow nasal oxygen therapy was not inferior to non-invasive ventilation with similar intubation rates of 14% in both groups [44].

To sum up the management of postoperative patients [Fig. 1], standard oxygen is sufficient after a surgical procedure, even after major ones such as abdominal or cardiothoracic surgery (e.g.*,* in patients with expected intubation rates around 5%). However, the standard oxygen should probably be switched to high-flow nasal oxygen in patients with hypoxemia (e.g.*,* patients with expected intubation rates around 10–15%) and further to non-invasive ventilation in patients with respiratory failure with hypoxemia and high respiratory rate and/or clinical signs of respiratory distress (e.g.*,* in patients with expected intubation rates reaching 50%).

## Conclusion

Although high-flow nasal oxygen seems to be an effective alternative to standard oxygen in patients with low risk of extubation failure in ICUs, the prophylactic use of non-invasive ventilation should be proposed as the first-line strategy of oxygenation in patients with high risk of failure. By contrast, standard oxygen seems sufficient in postoperative patients and high-flow nasal oxygen should be used in patients with hypoxemia.

Non-invasive ventilation may decrease the risk of intubation in postoperative patients with respiratory failure, but it could increase the risk of death by delaying reintubation in patients with post-extubation respiratory failure in the ICU.

## Funding

None.

## Conflicts of Interest

The authors declare that they have no known competing financial interests or personal relationships that could have appeared to influence the work reported in this paper.
